# Certolizumab enhances spinal cord injury recovery in rats through inhibition of the TNF-α signaling pathway and neuronal apoptosis

**DOI:** 10.1007/s10787-025-01674-5

**Published:** 2025-02-26

**Authors:** Ozan Küçükatalay, Çağlar Türk, Çevik Gürel, Gökçe Ceren Kuşçu, Mustafa Eren Yüncü, İnanç Karakoyun, Murat Akşit, Onur Sarıkaya, Ali Karadağ, Mahmut Çamlar

**Affiliations:** 1 Erzincan Mengücek Gazi Training and Research Hospital, Department of Neurosurgery, Erzincan, 24180 Turkey; 2 University of Health Sciences, Izmir City Hospital, Department of Neurosurgery, Izmir, 35100 Turkey; 3 Harran University, Faculty of Medicine, Department of Histology and Embryology, Şanlıurfa, 63050 Turkey; 4 Ege University, Faculty of Medicine, Department of Histology and Embryology, Izmir, 35100 Turkey; 5 University of Health Sciences, Izmir City Hospital, Department of Biochemistry, Izmir, 35100 Turkey; 6 University of Health Sciences, Izmir Tepecik and Education Hospital, Department of Biochemistry, Izmir, 35100 Turkey

**Keywords:** Apoptosis, Certolizumab, Neuroinflammation, Spinal cord injury, TNF-α

## Abstract

**Objective:**

Spinal cord injury (SCI), which is characterized by motor and/or sensory dysfunction, presents a significant health challenge resulting from mechanical trauma. Secondary injury, which follows the mechanical trauma and is driven by factors such as inflammation, plays a critical role in the SCI pathophysiology. Scientific evidence indicates that treatment strategies aimed at modulating inflammation during the acute phase of SCI alleviate the seconder injury. In this regard, the present study seeks to evaluate the effectiveness of certolizumab, a monoclonal antibody targeting TNF-α that is widely used in the treatment of various inflammatory diseases, in a SCI model.

**Methods:**

In this study, Control, Trauma, and Trauma + Certolizumab groups were established, each comprising eight male rats. One hour after SCI induction, rats in the Trauma + Certolizumab group were administered 10 µg Certolizumab dissolved in saline intraperitoneally, while rats in the Control and Trauma groups received an equivalent volume of saline. After Modified Tarlov Scoring was performed on the seventh day of the experiment, all rats were sacrificed. The effects of certolizumab on neuroinflammation and apoptosis in the SCI model were evaluated using histological, biochemical, and molecular analyses of blood and tissue samples obtained from the rats.

**Results:**

Certolizumab downregulated the expression of TNF-α, NF-κB, and IL-6. In addition, as evidenced by the TUNEL assay, Caspase-3 expression (an apoptotic marker), and Modified Tarlov Score results, certolizumab effectively suppressed inflammation-induced neural apoptosis and alleviated locomotor deficits.

**Conclusion:**

Certolizumab treatment exerts a neuroprotective effect against secondary damage in SCI through the inhibition of neuroinflammation and apoptosis.

## Introduction

Spinal cord injury (SCI), which affects over 27 million people worldwide, is a significant health issue characterized by motor and sensory impairments, or even paralysis, resulting in a reduced quality of life and imposing substantial economic and healthcare burdens on society (Hu et al. 2023; Huang et al. 2023; Fan et al. 2024). Since effective treatments are not yet available and current management primarily addresses symptoms, individuals with SCI continue to experience a reduced quality of life and higher mortality rates, despite advances in healthcare and specialized rehabilitation. Therefore, there is significant interest in developing novel therapeutic approaches to prevent or mitigate the impact of SCI (Lund et al. 2022). On the other hand, a comprehensive understanding of SCI pathophysiology and the identification of molecules targeting the biological phenomena involved in the pathophysiological cascade are crucial steps toward developing effective new therapeutic approaches for SCI (Lima et al. 2022).

The pathophysiology of SCI involves a complex and rapidly progressive process with acute and chronic phases (Wang et al. 2023). The acute phase of this process includes (i) the primary injury stage marked by nerve tissue destruction and ischemia following mechanical trauma such as motor vehicle accidents or falls from heights, and (ii) subsequent secondary injury stage defined by neurotransmitter-induced excitotoxicity, oxidative stress, and neuroinflammation (Müller et al. 2023). The stage where extensive scar formation and the progression of cystic cavities are observed in the injured spinal cord is the chronic phase of SCI pathophysiology (Alizadeh et al. 2019; Müller et al. 2023). Because excessive neuroinflammation leads to pathological changes such as neuronal cell necrosis, neuronal cell apoptosis, demyelination, Wallerian degeneration, and vascular endothelial cell damage, the most critical stage for spinal cord injury repair is the secondary injury stage where neuroinflammation is triggered (Huang et al. 2023; Fan et al. 2024). Hence, addressing the pathological damage resulting from neuroinflammation related secondary injury remains the central focus and challenge in SCI treatment (Zhang et al. 2021; Huang et al. 2023; Fan et al. 2024).

Cytokines secreted from activated astrocytes and microglia following primary injury play crucial roles as key actors in recruiting immune cells such as neutrophils and macrophages to the injury site and initiating the neuroinflammation process (Lukacova et al. 2021). Among these cytokines that contribute to the pathophysiology of SCI, one of the most extensively studied is tumor necrosis factor-alpha (TNF-α) (Esposito and Cuzzocrea 2011; Lund et al. 2022, 2023). In fact, scientific evidence point out that TNF-α is the most rapidly secreted proinflammatory cytokine after neuronal injury and that this cytokine functions as the initiator of Wallerian degeneration in injury site (Stoll et al. 2002; Esposito and Cuzzocrea 2011). In addition, several studies have reported that TNF-α triggers oxidative stress and apoptosis in SCI through activation of the NF-κB signaling pathway (Lund et al. 2023) and interleukin secretion (Xu et al. 1998). Furthermore, a variety of previous experimental studies using anti-TNF-α agents have demonstrated that inhibition of TNF-α in SCI mitigates injury and promotes locomotor functional recovery by inhibiting oxidative stress and neuronal apoptosis (Xu et al. 1998; Esposito and Cuzzocrea 2011; Wang et al. 2022; Lund et al. 2023). This literature data points that inhibition of TNF-α activity is an effective strategy for the treatment of acute-phase SCI (Esposito and Cuzzocrea 2011; Wang et al. 2022).

Certolizumab is a polyethylene glycol (PEG)-conjugated recombinant form of a humanized anti-TNFα monoclonal antibody that selectively neutralizes both soluble and membrane-bound TNF-α activity (Wang et al. 2024). Unlike other anti-TNF agents, certolizumab lacks the Fc portion of the IgG antibody, thus it does not induce complement-dependent or antibody-dependent cell-mediated cytotoxicity. In addition, certolizumab’s impact on immune cells differs; it does not trigger granulocyte degranulation or induce apoptosis in peripheral blood lymphocytes or monocytes, but instead leads to non-apoptotic cell death in these cells (Deeks 2016). The use of certolizumab has been approved by the U.S. Food and Drug Administration for the treatment of several inflammatory conditions including rheumatoid arthritis, Crohn’s disease, ankylosing spondylitis, psoriatic arthritis, and axial spondyloarthritis (Curtis et al. 2019). Furthermore, it has been reported that certolizumab may exert a protective effect in an experimental cerebral ischemia–reperfusion model by inhibiting the release of proinflammatory cytokines and microglial activation (Wang et al. 2024). On the other hand, the potential therapeutic effects of certolizumab in SCI have not yet been fully clarified. Considering the significant role of TNF-α in SCI, this research is poised to shed light on certolizumab’s potential therapeutic impact on SCI, particularly focusing on modulating neuroinflammatory response neuronal apoptosis, and oxidative stress.

## Materials and methods

### Ethical approval and experimental animals

The experimental protocol was evaluated and approved by Ege University, Local Ethics Committee for Animal Experiments (Approval No/Approval Date: 2016–085/28 December 2022). In accordance with ethical approval, 24 adult male Sprague Dawley rats weighing between 200 and 250 g were used in present study. Rats were caged at 24 °C ± 1 °C and had ad libitum access to food and water under a natural light/dark cycle. The number of animals and the care principles of the animals were determined in accordance with Russell and Burch's 3R principles (replacement, reduction, and refinement) (Tannenbaum and Bennett 2015; Hubrecht and Carter 2019).

### SCI modeling and experimental design

The rat SCI model was created following previously described methods (Xu et al. 1998; Camlar et al. 2019; Türk et al. 2024). Briefly, rats underwent dorsal laminectomy (T8-10) under combined ketamine (5 mg/kg; Ketalar, Pfizer, Istanbul, Turkey) and xylazine (10 mg/kg; Rompun, Bayer, Istanbul, Turkey) anesthesia. The paravertebral muscles and skin were sutured after this procedure in Control group (*n* = 8). In the Trauma (*n* = 8) and Trauma + Certolizumab (*n* = 8) groups, SCI was induced dropping a 10 g rod fell freely from the height of 2.5 cm. 60 min after the SCI induction, 10 µg of certolizumab was administered to the Trauma + Certolizumab group in accordance with the literature (Kosekli et al. 2019).

After experimental procedures, rats in each group were placed back in their cages separately. Paracetamol was added to the rats’ drinking water to provide analgesia. Daily bladder massage was performed on SCI-induced rats to prevent bladder glob formation.

### Evaluation of locomotor function

Hind limb motor function was classified according to Modified Tarlov Score (Camlar et al. 2019; Zhou et al. 2024) as follows: 0 (no voluntary hind limb function), 1 (poor hind limb motor function), 2 (joint motion present, but fails to stand), 3 (stands and walks), and 4 (complete recovery). The absence of muscle tonus and contractions was defined as paraplegia.

Locomotor function assessments were performed at 1 h before the SCI induction and 24, 48 h and the 7 days after SCI by three different investigators blinded to each other (Xia et al. 2024).

## Sample collection

After last locomotor function assessments, rats were anesthetized with combined ketamine (40 mg/kg; Ketalar, Pfizer, Istanbul, Turkey) and xylazine (4 mg/kg; Rompun, Bayer, Istanbul, Turkey). After anesthesia, rats’ previous incisions were reopened, and the spinal cord between T7 and T11 was removed as a en-block. Fresh tissue samples of four randomly selected animals from each groups were collected for real-time PCR and western blotting analysis. These samples were placed in sterile cryovials without fixation and stored at − 80 °C until analysis. In addition, tissue samples of other rats were collected for histological assessments and fixed in 4% paraformaldehyde for 48 h.

After spinal cord resection, quickly 1 ml blood samples were taken from the rats’ hearts for biochemical analyses. Blood samples were centrifuged at 1500 g for 10 min to separate the plasma and these plasma samples were stored at − 20 °C until analysis.

### Biochemical assessments

The plasma levels of total antioxidant status (TAS) and total oxidant status (TOS) were measured using commercial assay kits (Rel Assay Diagnostics, Gaziantep, Turkey) on the same autoanalyzer (Beckman Coulter Inc., CA, USA) (Erel 2005). TAS results were reported in mmol Trolox Eq/L and TOS results in μmol H_2_O_2_ Eq/L. The oxidative stress index (OSI) values were calculated using the formula 100 × [TOS (μmol H_2_O_2_ Eq/L)/TAS (mmol Trolox Eq/L)]. Results were expressed in arbitrary units (AU) (Erel 2004).

In addition, levels of IL-6 and TNF-α in the same plasma samples were measured using ELISA method with commercial test kits (Shanghai Sunred Biological Technology Co. Ltd, Shanghai, China) (Ok et al. 2020).

### Histological process and histochemical staining

Tissue samples were collected for histological assessments and fixed in 4% paraformaldehyde for 48 h. These samples were then dehydrated using a series of increasing alcohol concentrations and cleared with xylene. Following this, spinal cord samples were embedded in paraffin blocks. Cross sections, 5 µm thick, were obtained from these paraffin blocks and cleaned with xylene for histological assessments (Kuşçu et al. 2022).

Haematoxylin and eosin (HE) staining was performed for the evaluation of general histological parameters and detection of tissue damage. Briefly, tissues were deparaffinized with xylene and then incubated in distilled water for 5 min for rehydration. The tissue sections removed from distilled water were stained with hematoxylin dye for 3 min for nuclear staining purposes. Subsequently, to wash away excess dye and mordant the stain, the sections were sequentially incubated in running water and acid alcohol for approximately 5 min each until they turned pink. After rinsing in running water to remove acid alcohol, the tissue sections were incubated in ammoniated water until they turned blue. Following these steps, the tissues were incubated in distilled water for 5 min and then subjected to eosin staining for 1 min. Tissues stained with hematoxylin and eosin were passed through increasing series of alcohol (80%, 95%, and 100%, respectively). Following this stage, the sections were cleared with xylene and mounted with Entellan for examination under a light microscope (Gunusen et al. 2022).

### Immunohistochemical staining and Image J analysis of stained tissues

To block endogenous peroxidase activity, tissues were treated with a 10% hydrogen peroxide (H_2_O_2_) solution (Sigma Aldrich, Inc., St. Louis, Missouri, USA) for 30 min. Subsequently, sections were incubated with Super Block solution (Scytec Consulting Inc., Greenwood Village, Colorado, USA) for 1 h at room temperature. Following this, the sections were rinsed with PBS and then exposed to primary antibodies (IL-6 and NF-κB; Santa Cruz, California, USA) diluted at 1:200 for 24 h at + 4 °C. After primary antibody incubation, sections were sequentially treated with biotinylated secondary antibody (Scytec Consulting Inc.) and horseradish peroxidase (HRP)-conjugated streptavidin (Scytec Consulting Inc.). Finally, DAB staining was performed on the sections, followed by counterstaining with Mayer Hematoxylin (Merck, Germany) (Gurel et al. 2019).

The images were generated using a light microscope (Olympus; Tokyo, Japan). Three randomly selected slides, each containing ten different fields of the ventral horn area, were evaluated at a magnification of 40×. Ten specific regions were individually scrutinized, and the positive areas were quantified as a percentage of the total area. Image analysis was performed using the ImageJ 1.46 software. All tissue components except the background were identified, and the stained regions were recorded in an Excel spreadsheet. To isolate only the stained regions, the color slider was adjusted until only the immunohistochemistry (IHC) stained areas were highlighted and documented without altering the brightness slider. Subsequently, the integrated density values of the IHC stained regions were normalized to the integrated density values of the entire area. The resulting ratio of the immunostained area to the total area was graphically depicted (Grishagin 2015).

### ***Terminal deoxynucleotidyltransferase dUTP nick end labeling (TUNEL) analysis***

TUNEL analysis was conducted to assess apoptosis (Gurel et al. 2019) in spinal cord tissues from experimental groups. The apoptotic index (AI) for all groups was determined using the ApopTag® Peroxidase In Situ Apoptosis Detection Kit (Merck, Germany), following the kit’s instructions. AI was calculated based on the count of TUNEL-positive cells in photomicrographs of tissue sections subjected to the TUNEL assay. The counting process was independently repeated by three histologists who were blinded to each other’s evaluations, and the recorded numbers were averaged to determine the AI (Chang et al. 2014; Gurel et al. 2019).

### Western blotting (WB) analysis

For WB analysis, total proteins were extracted from the whole spinal cord tissues, followed by homogenization and sonication of the samples. After centrifugation at 10,000 g for 10 min, protein concentrations in the supernatant were determined using a standard bicinchoninic acid (BCA) assay. The samples were then heated at 95 °C for 5 min. Subsequently, they were subjected to SDS polyacrylamide gel electrophoresis under standard conditions and transferred overnight at 4 °C onto a polyvinylidene difluoride (PVDF) membrane in a buffer containing 0.2 mol/L glycine, 25 mM Tris, and 20% methanol. To prevent non-specific binding on the membranes, they were treated with 5% non-fat dry milk. Following blocking, the membranes were incubated overnight at 4 °C with primary antibodies against TNF-α (dilution 1:1000; Santa Cruz; California, USA) and β-actin (dilution 1:1000; Santa Cruz; California, USA). After washing, the membranes were incubated for 1 h at room temperature with a secondary antibody (dilution 1:3000, Thermo Scientific; Waltham, Massachusetts, USA). Visualization of protein binding was achieved using the chemiluminescence-based Super Signal CL HRP Substrate System (Thermo Scientific; Waltham, Massachusetts, USA), and the membranes were imaged using an ChemiDoc MP Imaging System (BioRad;California, USA). Antibody binding sites were identified as bands, and semi-quantitative analysis of these bands was performed using the Image J software package (NIH, Bethesda, MD, USA) (Chang et al. 2014).

### Real-time PCR analysis

Spinal cord samples weighing 50 mg each were mixed with 1 ml of TriPure Isolation Reagent containing guanidinium thiocyanate (Roche Applied Science, Penzberg, Germany) and homogenized using a glass-Teflon homogenizer. RNA isolation was then carried out following the instructions provided in the TriPure Isolation Reagent Kit protocol. Subsequently, 1 μg of RNA from each sample was utilized for cDNA synthesis. First-strand complementary DNAs (cDNAs) were synthesized using the Transcriptor First Strand cDNA Synthesis kit (Roche; Darmstadt, Germany) with total RNA. Real-time PCR analysis was conducted employing SYBR^®^ Green PCR Master Mix (ThermoFisher, Waltham, USA) and the LightCycler 480 system (Roche; Darmstadt, Germany) (Gurel et al. 2019). Relative ratios were determined using the 2^−ΔΔCt^ method with GAPDH as the reference gene, and each experiment was performed with three replicates (Rao et al. 2013). The primary sequences are provided in Table 1.

**Table 1:** The primers used for real-time PCR. F: forward primer; R: reverse primer

Gene	Primer sequences	References
TNF-α	F: 5’-ATGGGCTCCCTCTCATCAGT-3’ R: 5’-AAATGGCAAATCGGCTGACG-3’	(Shen et al. 2021)
IL-6	F: 5’-TCTGGTCTTCTGGAGTTCCG-3’ R: 5’-AGCATTGGAAGTTGGGGTAGG-3’	(Shen et al. 2021)
NF-κB	F: 5’-TGCCGAGTAAACCGGAACTC-3’ R: 5’-CAGCCAGGTCCCGTGAAATA-3’	(Shen et al. 2021)
Caspase-3	F: 5ꞌ-TACTCTACCGCACCCGGTTA-3ꞌ R: 5ꞌ-CGCGTACAGTTTCAGCATGG-3ꞌ	(Shen et al. 2021)
GAPDH (House Keeping)	F: 5’- AGACAGCCGCATCTTCTTGT-3’ R: 5’-CTTGCCGTGGGTAGAGTCAT-3’	(Shen et al. 2021)

### Statistical analysis

We utilized GraphPad Prism 5 software (GraphPad Software, Inc., La Jolla, CA) for statistical analysis. Following completion of all experiments, group comparisons were assessed using one-way analysis of variance (ANOVA) followed by post hoc Tukey tests. Data were presented as mean ± standard error, with statistical significance set at *p* < 0.05, *p* < 0.001, and *p* < 0.0001.

## Results

### Certolizumab improves locomotor function after SCI

**Fig. 1:**
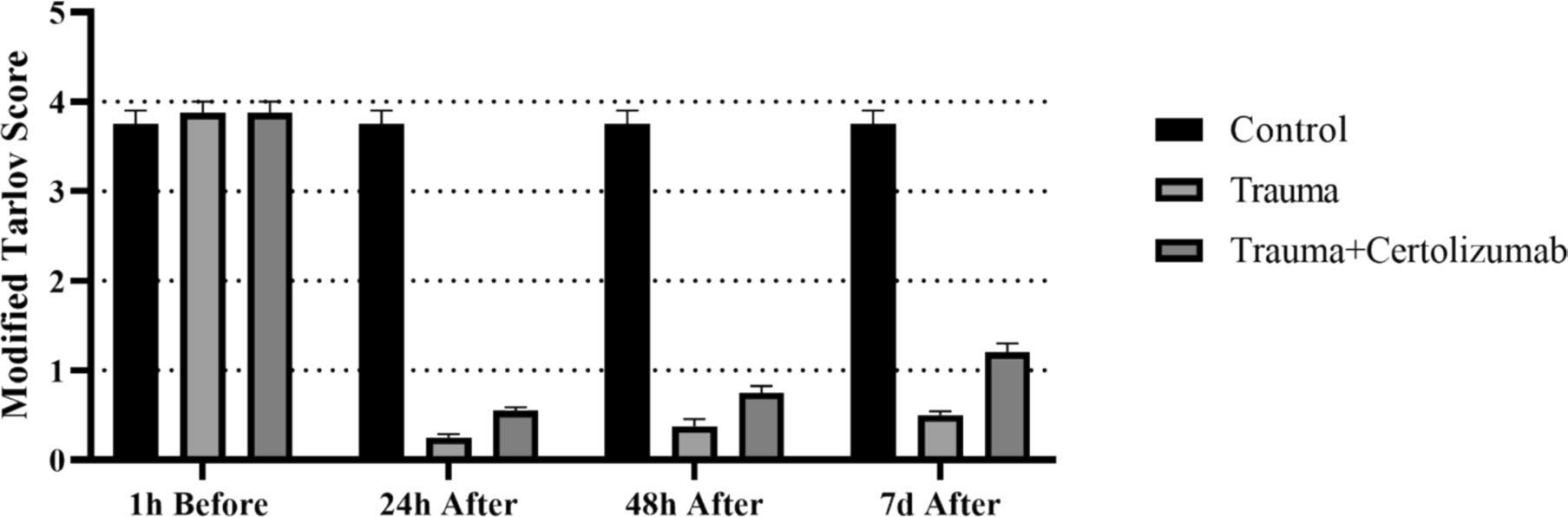
Tarlov Scores of rats in all experimental groups. No significant difference was observed between the groups in the Modified Tarlov Score evaluation conducted 1 h before SCI induction. However, at 24 and 48 h post-SCI, a significant decrease in the Modified Tarlov Score was noted in both the Trauma and Trauma + Certolizumab groups compared to the Control group. In addition, within the Trauma groups, the Modified Tarlov Scores at 24 and 48 h were significantly higher in the Trauma + Certolizumab group (*p* < 0.001). On the 7th day, while no significant difference was observed in the Modified Tarlov Score between the Control and Trauma groups, a significant increase was found in the Trauma + Certolizumab group (*p* < 0.05)

No significant difference was observed between the groups in the Modified Tarlov Score evaluation applied to 1 h before SCI induction (Fig. 1). In the evaluation at 24 and 48 h post-SCI, a significant decrease in Modified Tarlov Score was observed in both the Trauma and Trauma + Certolizumab groups compared to the control group. In addition, when comparing within the trauma and Trauma + Certolizumab groups, it was noted that the Modified Tarlov Scores at 24 and 48 h were significantly higher in the Trauma + Certolizumab group (*p* < 0.001). In the evaluation on the 7th day, while no significant difference was observed in terms of Modified Tarlov Score in the Control and Trauma groups, a significant increase was found in the Trauma + Certolizumab group (*p* < 0.05) (Fig. 1).

### Certolizumab alleviates SCI-associated oxidative stress

**Fig. 2:**
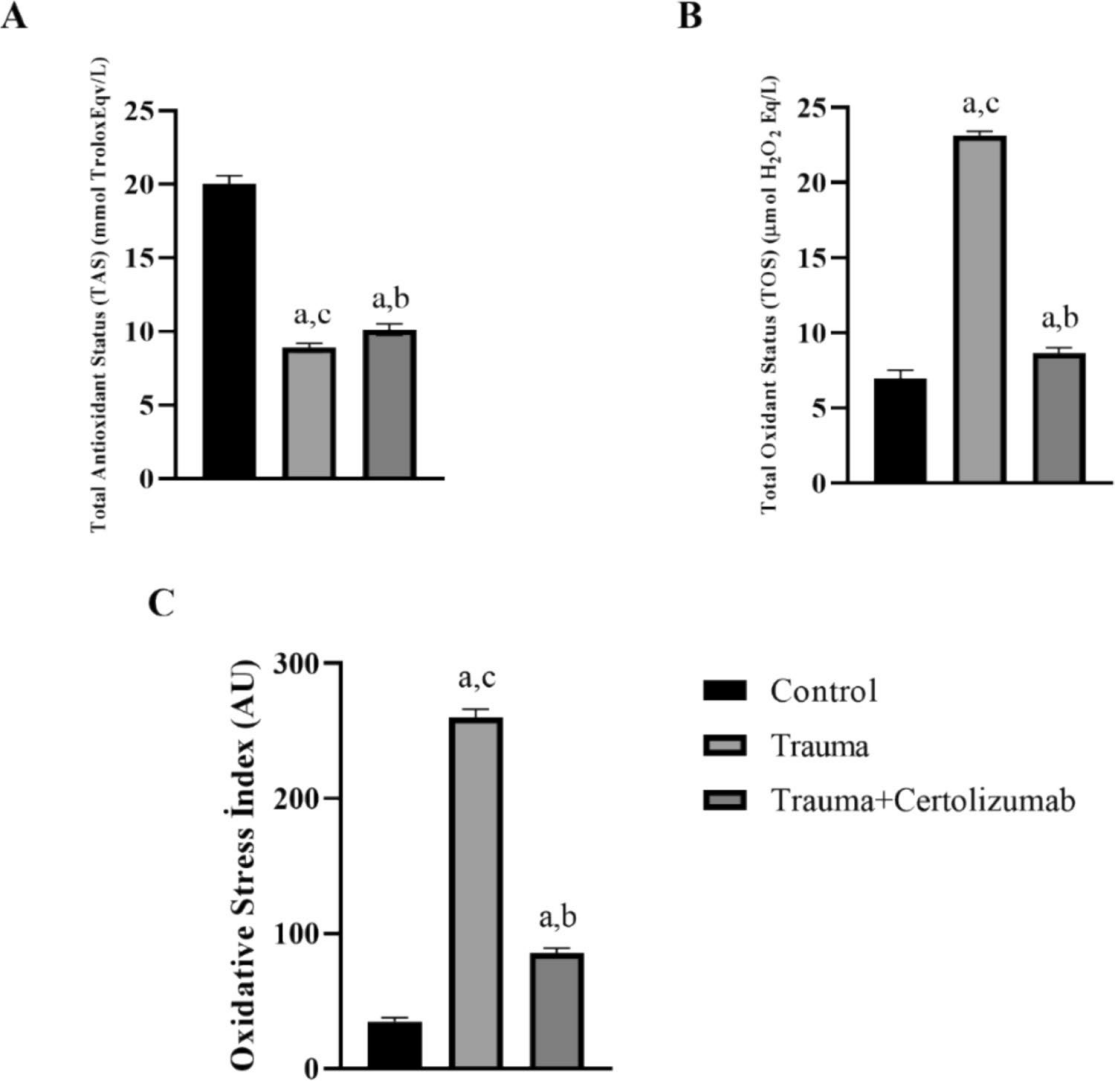
Graphs of total antioxidant status (TAS) (**A**), Total oxidative status (TOS) (**B**), and oxidative stress index (OSI) (**C**) analyses. A significant increase in TAS values and a significant decrease in TOS and OSI values were observed in the Trauma + Certolizumab group compared to the Trauma group (*p* < 0.05). a: significant change compared to the Control group, b: significant change compared to the Trauma group, c: significant change compared to the Trauma group Trauma + Certolizumab group

The findings obtained for total antioxidant status (TAS), Total oxidative status (TOS), and oxidative stress index (OSI) analyses are shown in Fig. 2.

Accordingly, it was determined that there was a significant increase in TAS values and a significant decrease in TOS and OSI values in the Trauma + Certolizumab Group compared to the Trauma group (*p* < 0.05).

### Certolizumab suppresses increased TNF-α mRNA and protein expression following SCI

**Fig. 3:**
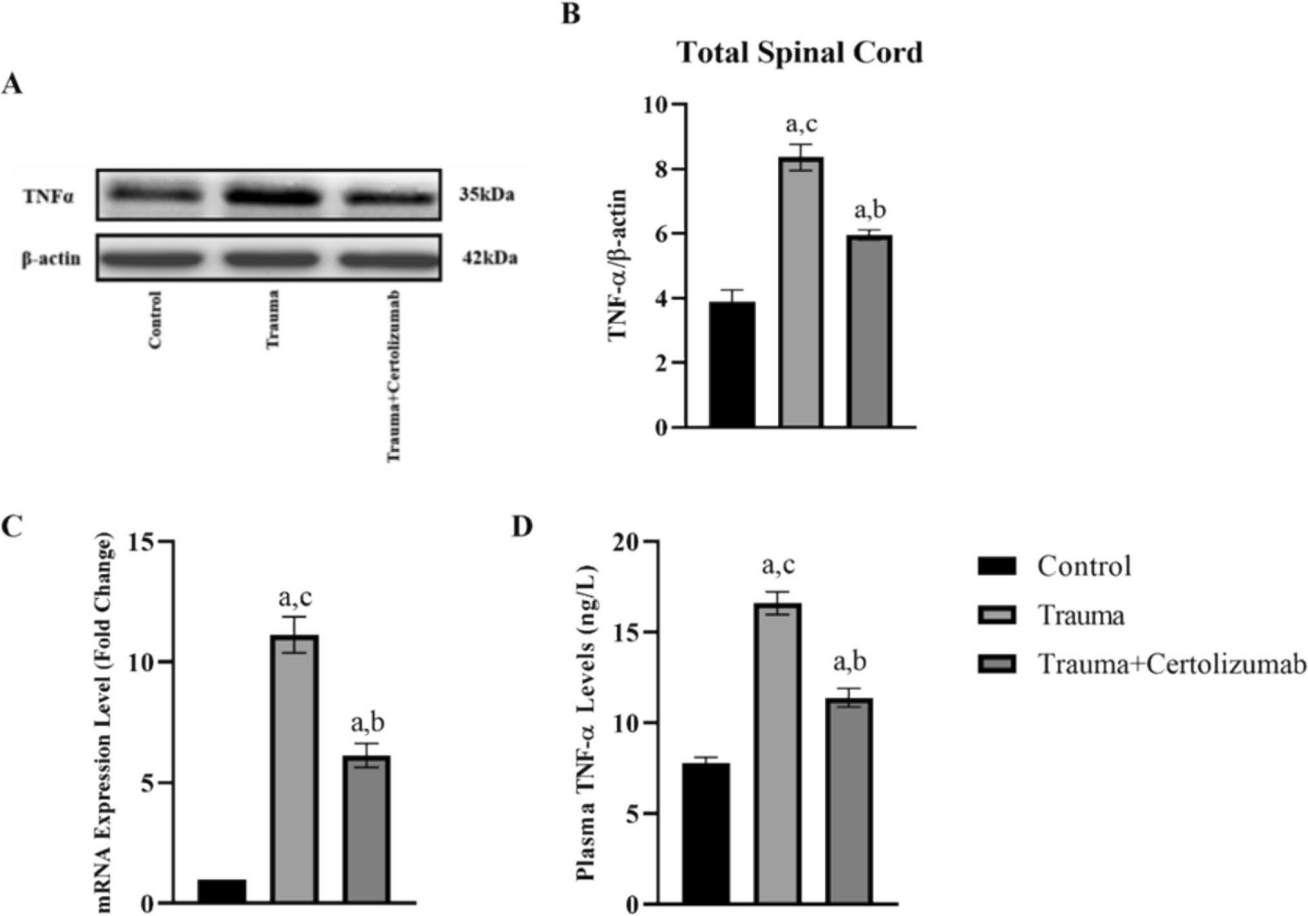
The graph depicts the ratio of bands representing TNF-α expression, obtained via western blot analysis in spinal cord tissue, normalized to β-actin (**A**). Graphic of the density ratios of TNF-α and β-actin bands obtained by western blot method for all groups in spinal cord tissue (**B**). Graphics of relative mRNA expression levels (**C**) and plasma levels of TNF-α (**D**). a: significant change compared to the Control group, b: significant change compared to the Trauma group, c: significant change compared to the Trauma group Trauma + Certolizumab group

The results of real-time PCR, WB, and ELISA analyses reveal that TNF-α gene and protein expression in the Trauma group increased significantly (*p* < 0.0001) compared to the Control group, whereas in the certolizumab treatment group, TNF-α mRNA and protein levels decreased significantly (*p* < 0.0001) compared to the Trauma group (Fig. 3).

### Certolizumab treatment attenuates histopathological changes associated with SCI

The spinal cord tissue samples from Control, Injury, and Injury + Certolizumab groups were examined for gray matter (substantia grisea, GM) and white matter (substantia alba, WM).

**Fig. 4:**
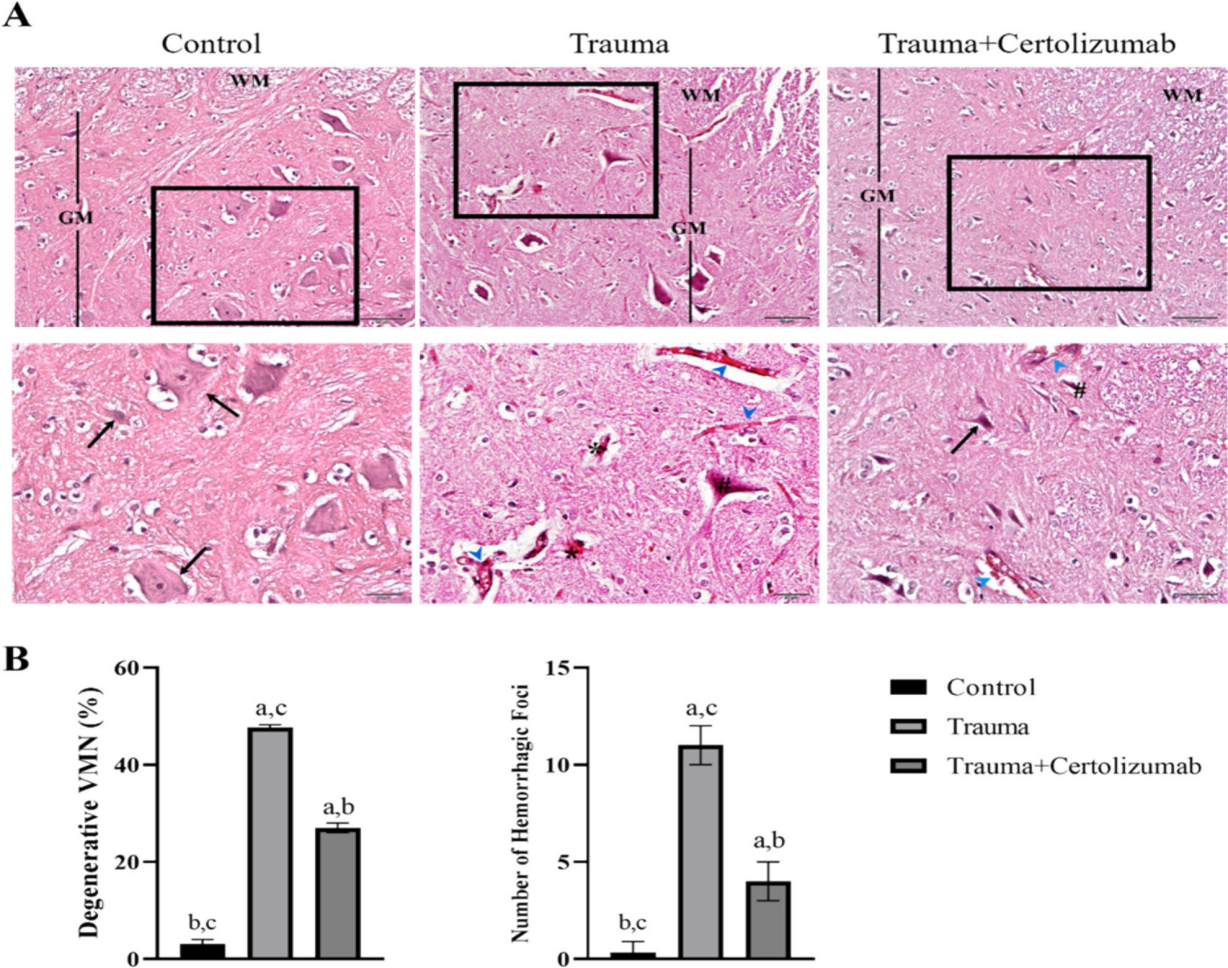
The figure shows the historical architecture of spinal cord samples (**A**) from all experimental groups and the results of degenerate ventral motor neuron (VMNs), and hemorrhagic focus counts (**B**) performed in these groups. The micrographs (upper panel 20X magnification, lower panel 40X magnification) introduce the histoarchitecture of spinal cord samples from all experimental groups (**A**). While black arrows indicate ventral motor neurons (VMNs) with normal morphology characterized by euchromatic nuclei, blue arrows represent hyperemic capillary vessels, *hemorrhagic areas, and #indicate degenerated VMNs. a: significant change compared to the Control group, b: significant change compared to the Trauma group, c: significant change compared to the trauma group Trauma + Certolizumab group, GM: gray matter, WM: white matter

In the spinal cord tissues of the Control group, the morphology of multipolar neurons located in the ventral horn and funicular neurons located in the dorsal horn appeared normal. Evaluation of the WM revealed normal axonal arrangement in both the anterior and posterior funiculi (Fig. 4).

In the assessment of spinal cord tissues from the Trauma group, histopathological changes such as hemorrhage and edema were observed within the GM, along with pathological alterations including axonal degeneration and necrosis in neurons located in the ventral and dorsal horn. Evaluation of the WM revealed particularly notable histopathological changes such as hemorrhage and edema within the anterior funiculus (Fig. 4).

In the histological evaluation of the Trauma + Certolizumab group, similar to the Trauma group, histopathological changes such as hemorrhage and edema were observed within the GM, along with pathological alterations including axonal degeneration and necrosis in neurons located in the ventral and dorsal horn. Evaluation of the WM revealed particularly notable hemorrhage and edema in the regions adjacent to the anterior horn of the anterior funiculus. However, it was noted that the histopathological changes observed in both GM and WM were significantly lower than in the Trauma group (*p* < 0.0001), and the histopathological changes observed in the spinal cord tissue samples in this group were significantly higher than the Control group (*p* < 0.0001). On the other hand, it was determined that the histoarchitecture of this group was closer to Control group than to Trauma group (Fig. 4).

### Certolizumab inhibits neuroinflammation via downregulation of IL-6 and NF-κB expressions

**Fig. 5:**
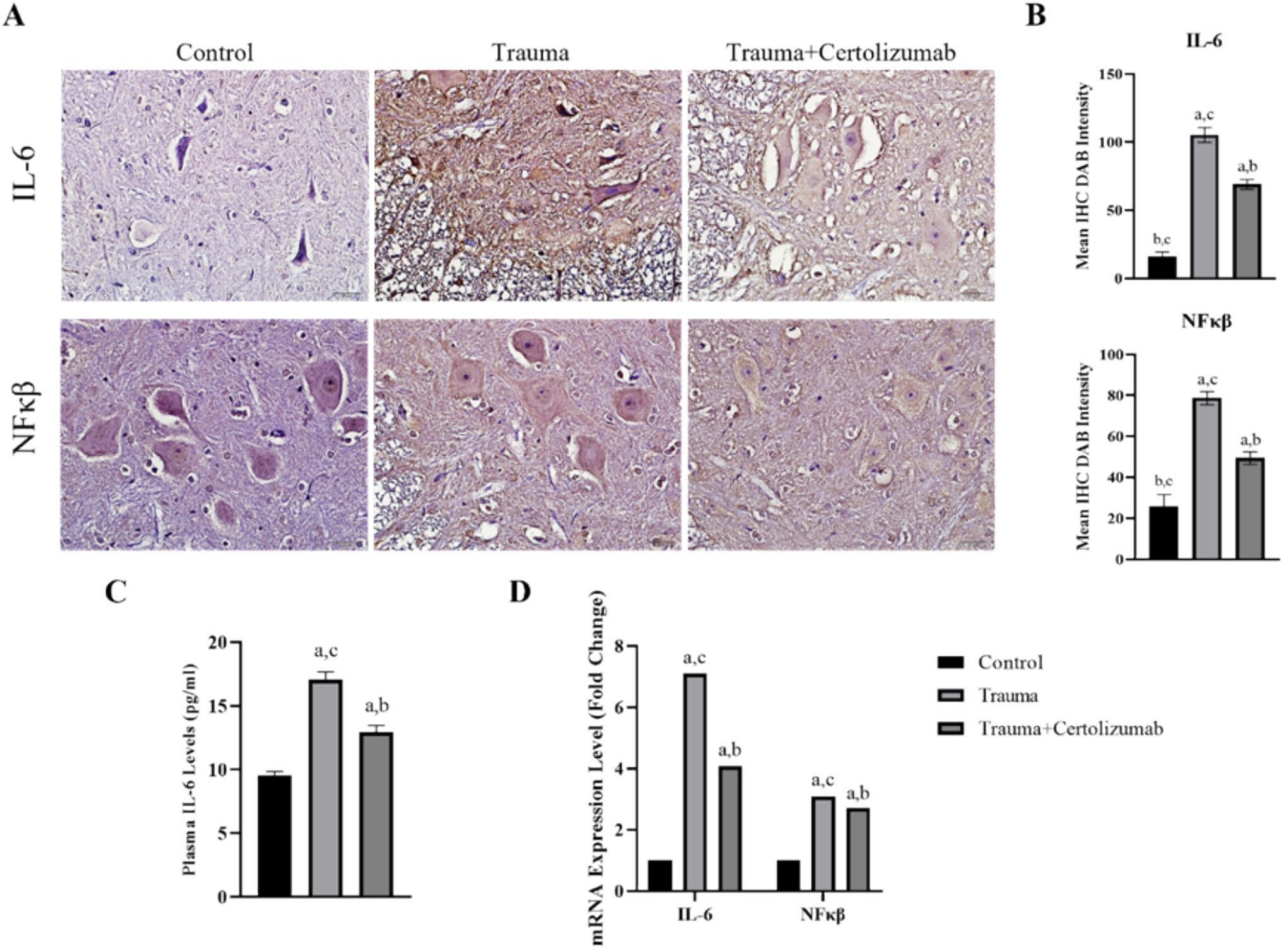
Spinal cord injury leads to an increase in neuroinflammation-associated IL-6 and NFκB protein (**A**, **B**) and mRNA (**D**) expression levels, while certolizumab alleviates this phenomena. In addition to this, certolizumab mitigates evaluated IL-6 plasma levels (**C**). a: Significant change compared to the Control group, b: significant change compared to the Trauma group, c: significant change compared to the Trauma + Certolizumab group

The conducted immunohistochemistry and real-time PCR analyses reveal a significant increase in neuroinflammation-associated IL-6 and NF-κB expressions [18] within the Trauma group compared to other groups (*p* < 0.0001) (Fig. 5). In addition, it is observed that the NF-κB protein pattern in the Trauma group is concentrated in the nucleus, while in other groups, NF-κB expression is predominantly cytoplasmic. In the group treated with certolizumab, both mRNA and protein expression levels of IL-6 and NF-κB show a significant decrease compared to the Trauma group (*p* < 0.0001).

### Certolizumab protects spinal cord neurons against SCI-induced neuronal apoptosis

**Fig. 6:**
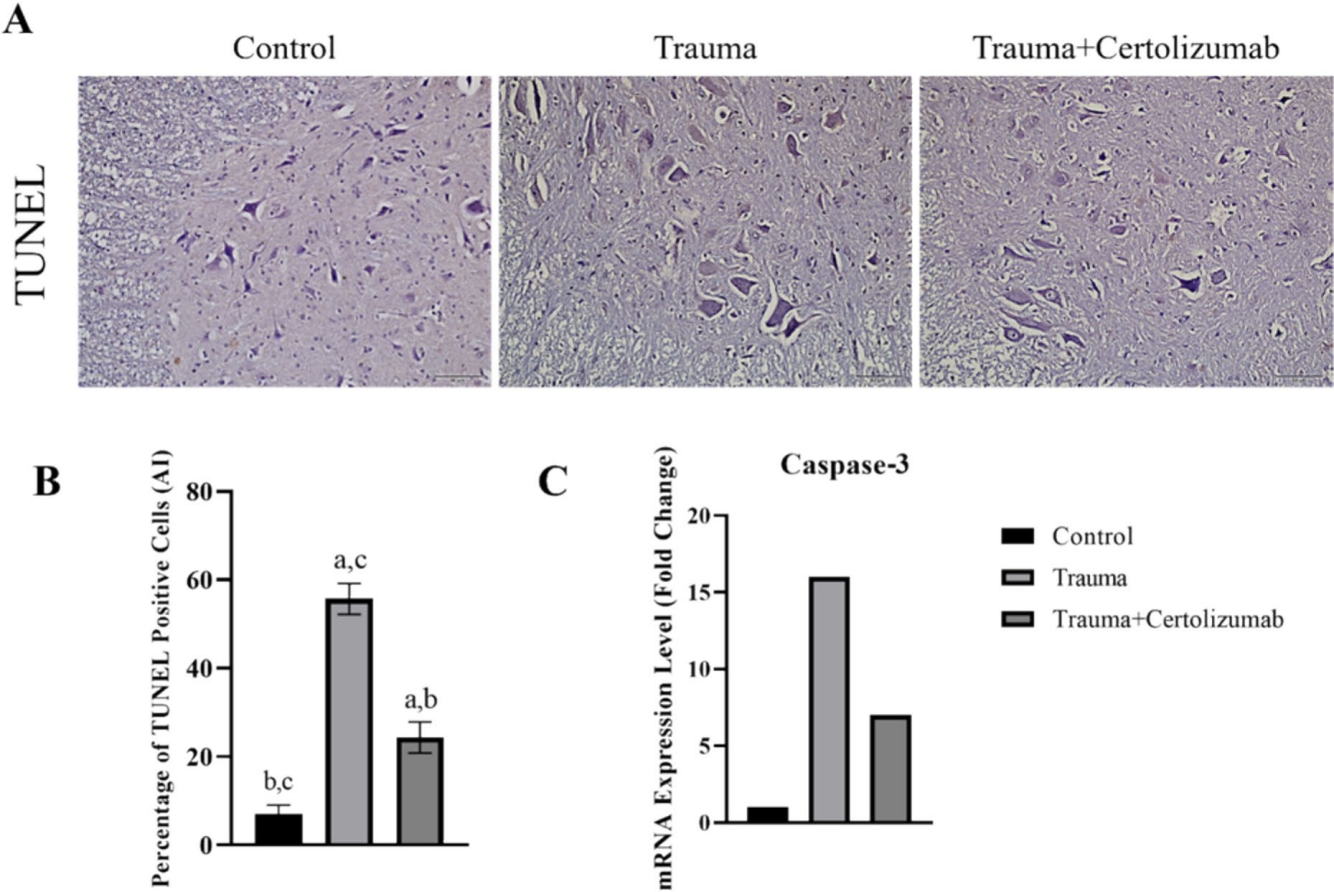
The figure shows apoptotic (TUNEL positive) cells (**A**) and the percentage of these cells in the groups (**B**) and the change in mRNA expression levels of Caspase-3, an important marker of apoptosis, in the groups (**C**). a: Significant change compared to the Control group, b: significant change compared to the Trauma group, c: significant change compared to the Trauma + Certolizumab group, AI: apoptotic index

In the evaluation of the Trauma and Trauma + Certolizumab groups, it was observed that the number of TUNEL-positive cells in the gray matter and the Caspase-3 mRNA expression in spinal cord tissue of these groups were significantly higher compared to the Control group (*p* < 0.0001). When these two groups were compared, it was determined that both the number of TUNEL-positive cells and Caspase-3 mRNA expression were significantly lower in the Trauma + Certolizumab group compared to the Trauma group (*p* < 0.0001) (Fig. 6).

It has been suggested that the mitigating effect of certolizumab on Caspase-3 mRNA expression, a significant marker of apoptosis [19], and the number of TUNEL-positive cells supports the notion that this drug may suppress neuronal death processes associated with neuroinflammation and oxidative stress.

## Discussion

Growing evidence suggests neuroinflammatory response that emerges during the acute phase of SCI and then becomes permanent contributes to long-term neurological dysfunction by triggering secondary damage (Larrea et al. 2023; Zhang et al. 2024). Moreover, findings from these studies have shown that TNF-α, the earliest proinflammatory cytokine released during the acute phase of SCI, regulates cellular events following spinal cord injury and plays a role in the onset and persistence of secondary damage (Streit et al. 1998; Stoll et al. 2002; Genovese et al. 2006; Esposito and Cuzzocrea 2011). Therefore, research findings targeting molecules such as TNF-α, known to play a role in the neuroinflammatory response following SCI, are crucial for developing new therapeutic approaches to mitigate secondary damage and treat SCI (Sterner and Sterner 2023). In this context, we explored the potential therapeutic efficacy of certolizumab, a monoclonal antibody targeting TNF-α, which is widely used in the treatment of various inflammatory diseases, in the present study. The results of our study provide compelling evidence that a single dose of certolizumab administered in the acute phase of SCI alleviated SCI-related histopathological changes and increased locomotor functional recovery. These findings are parallel with studies testing TNF-α blockers such as etanercept (Genovese et al. 2006, 2007), infliximab (Guven et al. 2010), and adalimumab (Börcek et al. 2015) in different SCI models. Indeed, these studies have shown that TNF-α inhibition alleviates histopathological changes such as edema and ischemia (Guven et al. 2010), reduces the number of TUNEL-positive (apoptotic) cells (Genovese et al. 2006), and leads to improvements in locomotor deficits (Guven et al. 2010; Börcek et al. 2015) caused by trauma. The histopathological and Tarlov Score findings of present study, which support these studies, indicate that TNF-α inhibition could be a potential therapeutic approach for SCI.

One of the key molecular mechanisms underlying the potential therapeutic efficacy of TNF-α inhibition in SCI may be the elimination of TNF-α’s master regulatory effect on other proinflammatory cytokines and modulators of inflammatory response such as IL-6 and NF-κB. In fact, it is known that IL-6 expression induced by TNF-α released during the acute phase of SCI leads to exacerbated neuroinflammation and increased tissue damage through the activation of macrophages and microglia (Mukaino et al. 2010; Croci et al. 2024). Furthermore, the inflammatory effector actions of TNF-α involve the activation of NF-κB, and neuroinflammatory responses triggered by activated microglia through the NF-κB pathway are key factors contributing to secondary injury (Xu et al. 1998; Chen et al. 2018). NF-κB subsequently increases the expression of proinflammatory genes, including acute-phase proteins, cellular adhesive molecules (CAMs), inducible nitric oxide synthase (iNOS), interleukins, proteases, and others, thereby sustaining and amplifying inflammatory activity (Xu et al. 1998). The results from the study’s immunohistochemical and biochemical analyses, which reveal a simultaneous decrease in TNF-α, IL-6, and NF-κB expression levels, strengthen the notion of the mechanisms associated with TNF-α inhibition’s potential therapeutic efficacy in SCI, as supported by existing literature.

Another molecular mechanism underlying the potential therapeutic efficacy of TNF-α inhibition in SCI may be the blockade of oxidative stress-mediated neuronal apoptosis. Indeed, a study on rat spinal cord explants introduced that TNF activation causes oxidative stress-mediated neuronal death in these cells through NF-κB-dependent iNOS expression, which leads to protein oxidation and nitration (Mir et al. 2009). In addition, it has been reported that after injury, the accumulation of oxidative products such as superoxide anion, hydroxyl radical, and peroxynitrite activates Caspase-3, which triggers apoptosis in spinal cord motor neurons (Xu et al. 2005; Moon et al. 2012). Moreover, several studies have shown that an increase in the oxidative status index (OSI) is paralleled by an increase in the apoptotic index (AI), indicated by the number of TUNEL-positive cells (Xu et al. 2005; Guven et al. 2010; Gürkan et al. 2020; Kinali et al. 2021). Our study findings, consistent with the existing literature, suggest that TNF-α inhibition may have a neuroprotective effect in SCI by blocking the oxidative stress-induced Caspase-3-mediated apoptotic process. Also, as mentioned above, although similar results have been obtained with other anti-TNF-α agents, certolizumab is distinct in that it does not induce complement-dependent or antibody-dependent cell-mediated cytotoxicity (Tutuncu and Kavanaugh 2017).

Nonsteroidal anti-inflammatory drugs (NSAIDs) are commonly used for pain relief and reducing inflammation (Lambrechts and Cook 2020). Due to their potent anti-inflammatory properties, NSAIDs have been extensively studied in experimental spinal cord injury (SCI) models. Indeed, research has demonstrated that NSAIDs compounds such as ibuprofen and naproxen may help reduce tissue damage and promote recovery of motor function in SCI models (Hayta and Elden 2018). Nevertheless, long-term or high-dose use of NSAIDs can negatively affect the cardiovascular system by elevating blood pressure, causing fluid retention, and impairing renal function (Drożdżal et al. 2021). Furthermore, certain NSAIDs may heighten the risk of cardiovascular events, including heart attacks and strokes, presenting a significant concern for patients who already have pre-existing health conditions, such as SCI (Davis and Robson 2016). On the other hand, there is strong evidence that TNF-α inhibitor therapy reduces the incidence of cardiovascular events in patients with inflammatory diseases such as rheumatoid arthritis (Jacobsson et al. 2007). For example, a study published by Göğebakan and Çetin demonstrated that certolizumab treatment reduced the risk of cardiovascular events in patients with ankylosing spondylitis by increasing insulin sensitivity and improving lipid components such as high-density lipoprotein cholesterol (HDL-C) and triglycerides (TG) (Göğebakan and Yıldırım Çetin 2022). These findings suggest the potential of long-term certolizumab use in regulating the neuroinflammatory process in SCI. Despite this, the presence of case reports in the literature (Lazarewicz et al. 2018) indicating that certolizumab use may lead to cardiac events highlights the necessity for in-depth investigation into the potential of this drug in SCI.

In conclusion, certolizumab treatment may exert a neuroprotective effect against secondary damage in SCI through the inhibition of NF-κB- and IL-6-mediated neuroinflammation and Caspase-3-induced neuronal apoptosis. Therefore, this study is significant as it is the first to demonstrate the efficacy of certolizumab in SCI.

## Limitations of the study

The primary limitation of this study is the inability to create groups for comparing the efficacy of a single dose of certolizumab with repeated doses. Another significant limitation is the lack of groups for comparison with other anti-TNF-α agents. In addition, the exclusion of female animals from the experiments can be considered a drawback.

Furthermore, the absence of protein measurement using analyses such as immunohistochemistry, biochemistry, or western blot for Caspase-3 is another limitation. The main reason for these limitations is the insufficient financial support provided for conducting the study.

## Data Availability

The datasets generated during and/or analyzed during the current study are available from the corresponding author on reasonable request.
